# Terahertz Electromagnetic Fields (0.106 THz) Do Not Induce Manifest Genomic Damage *In Vitro*

**DOI:** 10.1371/journal.pone.0046397

**Published:** 2012-09-27

**Authors:** Henning Hintzsche, Christian Jastrow, Thomas Kleine-Ostmann, Uwe Kärst, Thorsten Schrader, Helga Stopper

**Affiliations:** 1 Institut für Pharmakologie und Toxikologie, Universität Würzburg, Würzburg, Germany; 2 Physikalisch-Technische Bundesanstalt (PTB), Braunschweig, Germany; 3 Helmholtz-Zentrum für Infektionsforschung, Braunschweig, Germany; IIT Research Institute, United States of America

## Abstract

Terahertz electromagnetic fields are non-ionizing electromagnetic fields in the frequency range from 0.1 to 10 THz. Potential applications of these electromagnetic fields include the whole body scanners, which currently apply millimeter waves just below the terahertz range, but future scanners will use higher frequencies in the terahertz range. These and other applications will bring along human exposure to these fields. Up to now, only a limited number of investigations on biological effects of terahertz electromagnetic fields have been performed. Therefore, research is strongly needed to enable reliable risk assessment.

Cells were exposed for 2 h, 8 h, and 24 h with different power intensities ranging from 0.04 mW/cm^2^ to 2 mW/cm^2^, representing levels below, at, and above current safety limits. Genomic damage on the chromosomal level was measured as micronucleus formation. DNA strand breaks and alkali-labile sites were quantified with the comet assay. No DNA strand breaks or alkali-labile sites were observed as a consequence of exposure to terahertz electromagnetic fields in the comet assay. The fields did not cause chromosomal damage in the form of micronucleus induction.

## Introduction

Terahertz electromagnetic fields are non-ionizing electromagnetic fields in the frequency range from 0.1 THz to 10 THz. In contrast to other frequency regions in the electromagnetic spectrum, terahertz electromagnetic fields have not been used extensively for applications in the past due to a lack of suitable generators and detectors. This “terahertz gap” was overcome during the last decade and technical applications are being developed. One application, which is often associated with terahertz electromagnetic fields, is the body scanner which is employed at security checkpoints, e. g. at airports. The currently employed devices are working with millimeter waves, but scanners working at around 0.1 THz are being developed. Other applications, like data transmission or medical imaging, are also being developed for the terahertz frequency region and are thought to be applied within the next decade. All of these applications involve exposure of the general public and require toxicological risk assessment [1], [2], [3].

Effects of electromagnetic fields in general have been investigated widely, however, the majority of the studies investigated radiofrequency electromagnetic fields used for mobile communication. There is consensus that high power electromagnetic fields cause heating which can be responsible for a variety of biological effects. Non-thermal effects at low power intensities were postulated but have not been proven consistently [4]. Investigations showed partly contradictory results on all biological levels, e. g. production of reactive oxygen species [5], [6], enzyme activity [7], [8], genotoxicity [9], [10] or cancer [11]. A number of review articles have dealt with effects of non-ionizing radiation [12], [13], [14].

In the terahertz region only a few dozen studies have been published [15], the major contribution coming from the project “THz Bridge”, which was initiated and funded by the European Union and concentrated on frequencies around 0.1 THz [16], [17], [18]. The report concluded that genotoxicity was only observed under specific circumstances, which was later reported as aneuploidy [19]. The recently reported mitotic disturbances [20] which are in general thought to develop into genomic damage in the form of micronucleus formation, might be in line with a potentially genotoxic effect of terahertz electromagnetic fields. However, other studies did not report micronucleus formation [18], [21]. It was reported that terahertz electromagnetic fields caused gene expression changes at low intensities, while the mechanism for this effect remains unclear at the moment [22], [23]. As expected, it was shown that high power terahertz electromagnetic fields lead to thermal effects in analogy to other frequency regions [24].

In the THz-Bridge project, leukocytes were mainly used as target cells. It is estimated that at 0.1 THz these electromagnetic fields can penetrate the human skin only a few hundred micrometer, and therefore may be able to reach small blood vessels. However, due to their higher chances of exposure, various cell types of the skin are an even more relevant target which had not been used for genotoxicity testing of electromagnetic fields around 0.1 THz before.

The aim of the current study was therefore to assess the potential of 0.1 THz electromagnetic fields, the frequency which had induced mitotic disturbances, for the induction of genotoxic effects in two types of human skin cells and in the human-hamster hybrid cell line in which the mitotic disturbances had been found.

## Materials and Methods

### Materials

Chemicals were bought from Sigma-Aldrich (Steinheim, Germany), PAA (Pasching, Austria) or Invitrogen Life Technologies (Darmstadt, Germany). FCS was purchased from Biochrom (Berlin, Germany). HaCaT cells were purchased from Cell Line Service (Eppelheim, Germany). HDF cells were purchased from Greiner BioOne (Frickenhausen, Germany). A_L_ cells were kindly provided by Prof. Dr. Ernst Schmid (Munich, Germany). These cells have previously been used in mobile phone exposure studies [25], [26]. Phosphate buffered saline (PBS) contained 8 g NaCl, 0.2 g KCl, 0.56 g Na_2_HPO_4_, and 0.2 g KH_2_PO_4_ dissolved in 1 l of demineralized water. Dabco solution contained 250 mg Dabco dissolved in 10 ml PBS and mixed with 90 ml glycerol.

### Cell Culture

HaCaT and HDF cells were cultured in DMEM medium (4.5 g/l glucose) and A_L_ cells were cultured in RPMI-1640 medium under regular cell culture conditions (37°C, humidified atmosphere, 5% CO_2_). The medium was supplemented with 10% FCS, 2 mM L-glutamine, and antibiotics. One day before the experiments, cells were seeded onto a circular area of 1.13 cm^2^ in the center of coded μ-dishes (ibidi, Martinsried, Germany) with the help of cell culture inserts (Flexiperm, Greiner BioOne, Frickenhausen, Germany). For the micronucleus test 40,000 cells and for the comet assay 60,000 cells were seeded. After the cells had attached to the dish bottom, the inserts were carefully removed from the dish. Cells with similar passage number were thawed for each replicate experiment.

### Exposure setup

**Figure 1:**
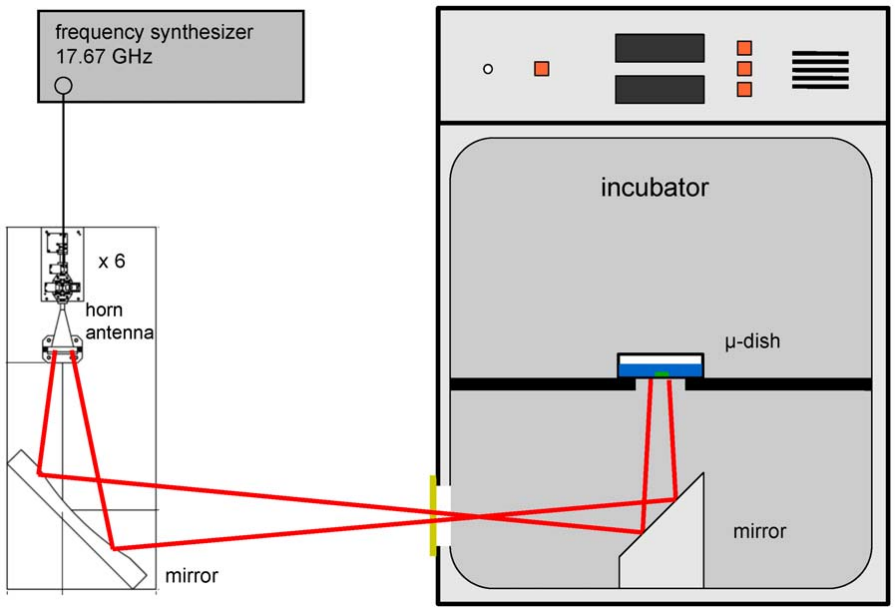
Scheme of the exposure set-up showing the exposure incubator and the source of the THz electromagnetic fields.

The cells in the coded μ-dishes were exposed from below with a collimated Gaussian beam at 0.106 THz in a modified incubator (NuAire NU-5100) at defined environmental conditions (Fig. 1). The cells were covered by approximately 4 mm of DMEM medium which absorbed the electromagnetic field passing through the cell monolayer completely.

The electromagnetic field originated from a frequency multiplier chain. A continuous wave signal at 17.67 GHz from a frequency synthesizer (Agilent E8257D) was sextupled in a Schottky multiplier. The wave was fed into a round corrugated horn antenna via a variable attenuator that allowed adjustment of the radiated power between 0 and 155 mW. In front of the antenna, the electromagnetic field was collimated to a beam width (full-width half-maximum) of 2 cm at the location of the μ-dishes using a parabolic reflector made from solid metal. The collimated electromagnetic field was coupled in via a thin transparent window at the side of the incubator. A second flat metallic mirror located at the bottom of the incubator was used to direct the electromagnetic field onto the μ-dishes from below. The μ-dish bottom foil as well as the incubator window made of plastic foil showed radiation transmission of more than 95% at 0.106 THz. The μ-dishes were positioned on a support made of Rohacell 71 HF, a low dielectric constant and low loss material (Evonik Industries, Germany) that left the bottom of the dishes free for exposure. To avoid standing waves due to refracted or scattered waves, the metallic walls within the incubator were covered with absorption foil. Temperature measurements performed in the μ-dishes during separate experiments indicated that exposure with power densities of 1 mW/cm^2^ yielded cell medium temperature increases of 0.2°C, which is in the range of the temperature regulation fluctuation of the incubator.

To guarantee appropriate exposure conditions, the environmental parameters within the incubator (temperature, humidity and CO_2_ content) were monitored continuously (Almemo Datalogger, Ahlborn, Holzkirchen, Germany). The empty field power density at the location of the μ-dishes was set traceable to the SI units [27] based on beam profile characterization using a dielectric fiber and measurement of the integrated radiant power in the beam using a calibrated photo-acoustic detector based on a closed air-cell with pressure transducer (Thomas Keating Power Meter, Thomas Keating Ltd., UK). Calibration was provided by Ohmic heating of a thin metal film within the detector head. The specified power densities represent averages over the exposure spot area with a diameter of 12 mm and have been calculated taking into account beam profile and radiant power.

### Exposure protocol

HaCaT and HDF cells were exposed to 0.106 THz electromagnetic fields with power densities between 0 mW/cm^2^ and 0.88 mW/cm^2^ for 2 h, 8 h and 24 h duration. For the sham exposure (0 mW/cm^2^), cells were placed in the exposure incubator at exactly the same place where the exposed cells were positioned; all conditions were the same except for the lack of the electromagnetic fields. In a separate set of experiments, cells were also exposed to higher power intensities of 2 mW/cm^2^. Before exposure, the appropriate power density was adjusted using the photo-acoustic detector at the location in the incubator where the μ-dish was placed. The specified power densities represent averages over the exposure area covered with cells with a diameter of 12 mm and an area of 1.13 cm^2^. The power dissipated in the investigated area can be calculated by multiplying the specified power densities by a factor of 1.13 cm^2^, e. g. a power density of 1 mW/cm^2^ corresponds to a power of 1.13 mW absorbed in the investigated sample area. Exact power density levels were set to (0.04±0.01) mW/cm^2^, (0.39±0.09) mW/cm^2^, (0.88±0.19) mW/cm^2^, and (1.96±0.45) mW/cm^2^. The given ranges indicate the power densities which the μ-dishes were exposed to (95% confidence intervals) including the uncertainties of the power adjustment and of signal fluctuations as obtained from a detailed uncertainty analysis. This analysis was performed according to the “ISO/IEC Guide 98-3:2008: Uncertainty of measurement – Part 3: Guide to the expression of uncertainty in measurement (GUM)”. Using the finite integration method, it has been determined numerically that empty field power densities of 2 mW/cm^2^ result in maximum specific absorption rates of 13.34 W/kg [28].

### Micronucleus test

The micronucleus test was originally developed in the 1970s [29] and was modified for the present investigation as described in this section. To be able to evaluate micronuclei in binucleated cells in HaCaT and A_L_ cells, cytochalasin B (3 µg/ml) was added directly after exposure or treatment and cells were further incubated for 24 h. Afterwards, the medium was removed, cells were washed with PBS and fixed in −20°C methanol for at least 1 hour. Then dishes were air-dried and stored until analysis. For the staining procedure anti-tubulin antibody solution was diluted 1∶50 and chromomycin A_3_ was dissolved in PBS containing 150 mM magnesium chloride, giving a final concentration of 100 mM of chromomycin A_3_. The cell layers on the dishes were incubated with anti-tubulin antibody solution for four hours at 37°C. Thereafter the dishes were rinsed with PBS and cells were incubated with chromomycin A_3_ for five minutes at room temperature. Finally, the preparation was mounted with Dabco mounting medium. Micronucleus analysis was performed at 400× magnification with a Nikon TE-2000-E microscope applying a regular FITC filter. The overall number of mononucleated (MN), binucleated (BN) and multinucleated (MuN) cells was analyzed as well as the frequency of binucleated cells containing micronuclei in 2,000 cells per dish. The following criteria had to be fulfilled by micronuclei:

- Staining similar to the main nuclei
- Location within the cytoplasm
- No overlap with the main nuclei
- Size approximately 1/16 to 1/3 of the main nuclei

For analysis of cell proliferation, the cytochalasin B proliferation index (CBPI) was calculated according to the formula CBPI = (1•MN+2•BN+3•MuN)/(MN+BN+MuN).

The procedure for the micronucleus test for the A_L_ cells was similar to the HaCaT cells with the exception of the staining, which was done using Gel-Green solution (1∶100 dilution, Biotrend, Cologne, Germany) for 3 minutes.

Because of negative effects on cell morphology, the HDF cells were not treated with cytochalasin B and micronuclei were evaluated in mononucleated cells. Cells were treated with 5-ethynyl-2′-deoxyuridine (EdU) 4 hours prior to the end of the post-exposure incubation period, i. e. the fixation point (final concentration 10 µM). The fixation procedure was similar to the one of the HaCaT cells. Then, cells were stained with bisbenzimide and cells which had incorporated EdU were visualized using a labeled azide (Click-it EdU kit, Invitrogen, Darmstadt, Germany). Micronucleus analysis followed the same criteria as before with the exception that CBPI proliferation analysis was replaced by the analysis of replication activity (EdU-incorporation, i. e. distinguishing EdU-positive and EdU-negative cells).

### Comet Assay

The comet assay is an electrophoresis-based method to quantify primary DNA damage, it was developed in the 1980s [30], [31]. For the present investigation it was modified according to following procedure. After exposure or treatment, cells were detached from the dish and 45 µl of the cell suspension were mixed with 160 µl of 0.5% low melting point agarose. 45 µl of this mixture were added to glass slides which had been covered with a layer of 1.0% high melting point agarose, and two slides were prepared from each exposure. The slides were stored in a cuvette containing lysis solution (2.5 M NaCl, 0.1 M EDTA, 0.01 M Tris and 1% Triton X-100, 10 g/l N-lauroylsarcosine sodium adjusted to pH 10 with NaOH) for at least 60 minutes. Then, the slides were incubated in electrophoresis solution (300 mM NaOH, 1 mM EDTA, pH>13.0) for 20 minutes, followed by an electrophoresis in the same solution (25 V, 300 mA, 20 minutes). Cells were neutralized for 5 minutes in Tris-solution and stained with a 1∶3 mixture Gel-Red/Dabco-solution (Biotrend, Cologne, Germany). 50 cells per slide were analyzed using a Nikon Labophot-2 microscope at 200× magnification and applying a regular TRITC filter. For quantification of the DNA damage, the percentage of DNA in the tail was measured using Komet 6 image analysis software (BFI Optilas, Dietzenbach, Germany).

### Statistics

All experiments were performed as three independent replicate exposure experiments. For the micronucleus test, 2,000 cells were evaluated for each replicate, resulting in a total of 6,000 cells. For the analysis of the cells exposed for 24 h, cell number was increased to 10,000 cells for each exposure and sham-exposure replicate (yielding a total of 30,000 cells) and to 6,000 cells per control replicate (yielding a total of 18,000 cells). For the comet assay, 50 cells were analyzed per slide and two slides per exposure condition were prepared, resulting in a total of 300 cells. The results of the exposed cells were compared to their sham-exposed controls using the Mann-Whitney-U-test. Differences were regarded as not significant when p≥0.05. Untreated controls and positive controls are presented as historical controls. This means that for these controls, cells were set up at various time points during the experimental series. The historical control approach allows controlling for time trends. Positive controls were treated with chemicals, whereas untreated cells were not treated and were not placed in the exposure incubator.

## Results

### Comet assay

**Figure 2:**
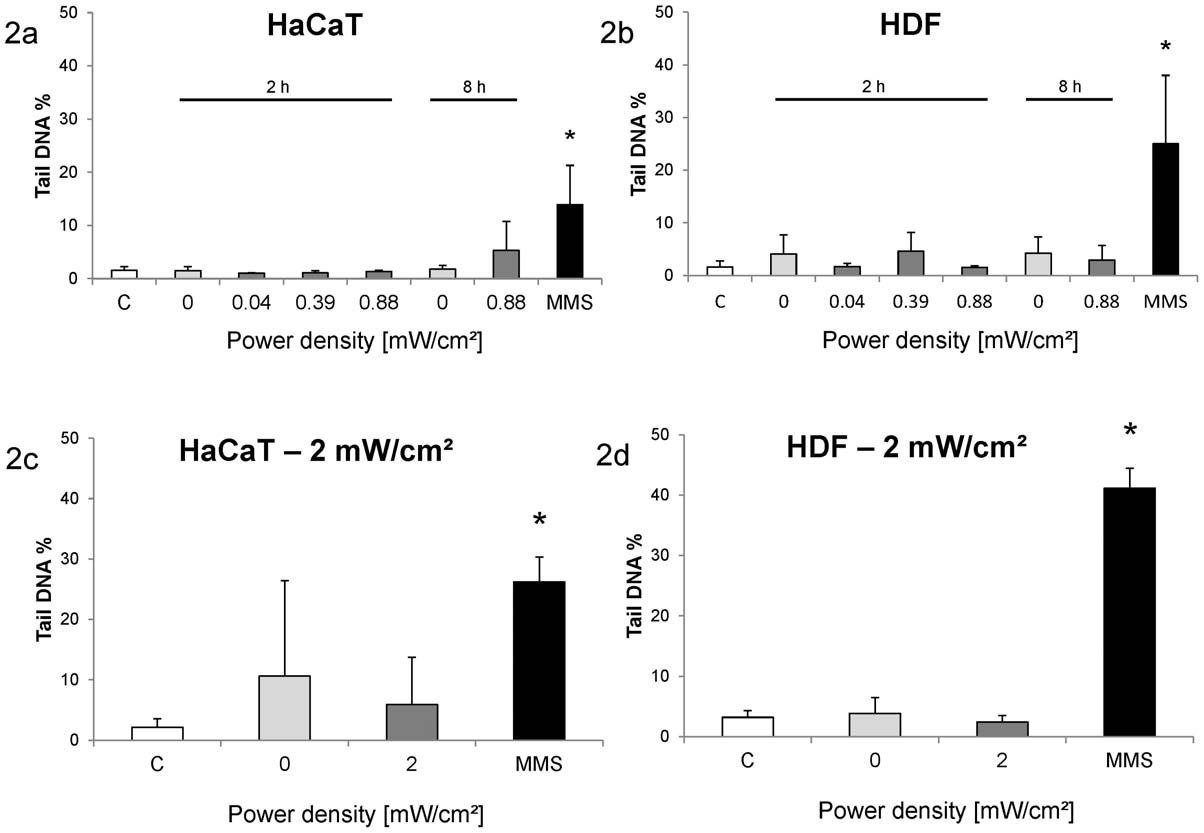
DNA migration (Tail DNA %) at different exposure conditions. Columns represent means and error bars represent standard deviations of at least three independent experiments (2×50 cells per replicate). Untreated controls (C) and positive controls (MMS) are presented as historical controls performed at different time points during the experiment series (12 independent replicates). Results are shown for HaCaT (2a, 2c) and HDF (2b, 2d) cells. MMS-treated cells showed significantly higher DNA migration compared to untreated cells (*, p<0.05).

DNA single and double strand breaks and alkali-labile sites were assessed using DNA migration in the comet assay. Cells were exposed with 0.04 mW/cm^2^, 0.39 mW/cm^2^ and 0.88 mW/cm^2^ for 2 h and with 0.88 mW/cm^2^ for 8 h (Fig. 2). After the short exposure, DNA migration in HaCaT cells was not increased in the exposed samples, whereas after the long exposure, the DNA in the tail region was increased in comparison to the sham-exposed sample (Fig. 2a). However, the difference was not statistically significant mainly due to a high variability in the exposed cells. The variability of the DNA damage values was slightly higher in the HDF cells, but no increase in the exposed cells compared to the sham-exposed cells was observed (Fig. 2b).

Positive controls were included as historical controls in order to demonstrate the test's ability to correctly detect DNA damage. Cells were treated with 150 µM methyl methanesulfonate (MMS) for 4 h. Both cell types exhibited significantly increased DNA damage as a consequence of this treatment (Fig. 2a & 2b). The value of the sham-exposed cells was similar to the untreated historical controls.

In a separate set of experiments, cells were additionally exposed to power intensities of 2 mW/cm^2^. An increase of DNA damage was not observed, neither in HaCaT cells (Fig. 2c) nor in HDF cells (Fig. 2d). Due to the large inter-experimental variability in the sham-exposed HaCaT cells, it might not have been possible to detect subtle changes in this case.

### Micronucleus test

**Figure 3:**
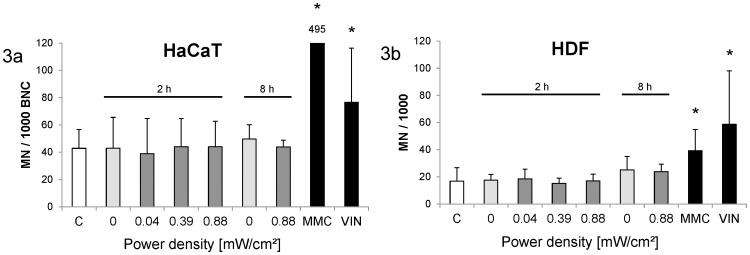
Micronucleus frequency after different exposure conditions. Columns represent means and error bars represent standard deviations of at least three independent experiments (2×1,000 cells per replicate). Untreated controls (C) and positive controls (MMC and VIN) are presented as historical controls performed at different time points during the experiment series (12 independent replicates). Results are shown as number of micronucleated cells per 1,000 binucleated cells for HaCaT (3a) and as number of micronucleated cells per 1,000 mononucleated cells for HDF (3b) cells. MMC-treated HaCaT cells showed a micronucleus frequency of 495±369 MN/1,000 BNC (Fig. 3a). MMC- and VIN-treated cells showed significantly higher micronucleus frequencies compared to untreated cells (*, p<0.05).

DNA damage on the chromosomal level was quantified with the help of the micronucleus test. Cells were again exposed with power intensities of 0.04 mW/cm^2^, 0.39 mW/cm^2^ and 0.88 mW/cm^2^ for 2 h and with 0.88 mW/cm^2^ for 8 h. In the HaCaT cells, increased micronucleus frequencies were observed neither in the 2 h nor in the 8 h exposure experiments (Fig. 3a). Micronucleus frequency in the HDF cells was generally lower, but again the exposed cells showed DNA damage levels similar to the sham-exposed cells (Fig. 3b).

As positive controls, cells were treated with the clastogen mitomycin C (MMC, 1.5 µM for 4 h) and the aneugen vinblastine (VIN, 5 µM for 4 h). Both led to clear micronucleus formation (Fig. 3a & 3b). The micronucleus frequencies of the sham-exposed cells were similar to the values of the untreated historical controls.

### Proliferation

**Figure 4:**
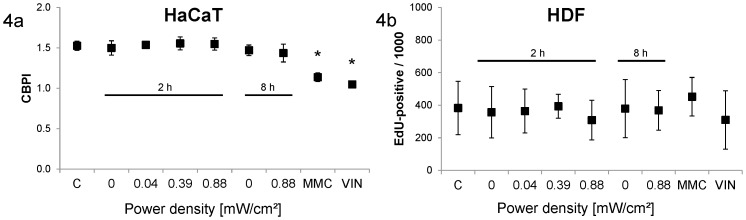
Proliferation rate after different exposure conditions. Columns represent means and error bars represent standard deviations of at least three independent experiments (2×1,000 cells per replicate). Untreated controls (C) and positive controls (MMC and VIN) are presented as historical controls performed at different time points during the experiment series (12 independent replicates). Results are shown as cytochalasin B proliferation index for HaCaT cells (4a) and as number of EdU-positive cells per 1,000 cells for HDF cells (4b). MMC- and VIN-treated HaCaT cells showed significantly lower proliferation indices compared to untreated cells (*, p<0.05).

Cell proliferation was quantified as a marker for cytotoxicity and to assess proliferation-related effects on DNA damage because micronuclei can only form when cells proliferate. For this assay the same preparations were used as for the micronucleus test, thus the exposure and treatment conditions were identical. In the HaCaT cells, proliferation was quantified as the cytochalasin B proliferation index, which was found to be unaffected by the electromagnetic field exposure (Fig. 4a). MMC and VIN treatment resulted in significantly decreased proliferation rates. In the HDF cells, proliferation was quantified as EdU-incorporation. No change in proliferation was observed for the exposed samples (Fig. 4b). MMC caused a slightly increased EdU incorporation and VIN led to a slightly decreased frequency of EdU-positive cells, but both changes were not statistically significant compared to the negative controls. The sham-exposed cells showed similar values as the untreated historical controls.

### Long term exposure

**Figure 5:**
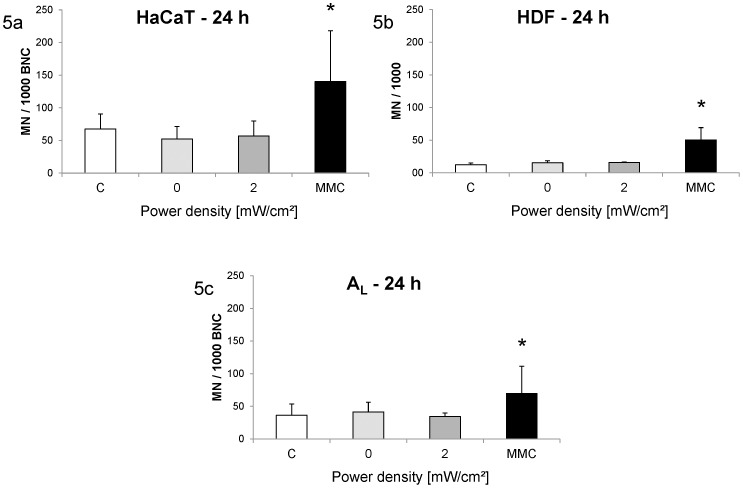
Micronucleus frequency after different exposure conditions. Columns represent means and error bars represent standard deviations of at least three independent experiments (at least 5×2,000 cells per replicate for exposed and sham-exposed samples; at least 3×2,000 cells per replicate for control samples). Results are shown as number of micronucleated cells per 1,000 binucleated cells for HaCaT (5a) and A_L_ (5c) cells and as number of micronucleated cells per 1,000 mononucleated cells for HDF (5b) cells. MMC-treated cells showed significantly higher micronucleus frequencies compared to untreated cells (*, p<0.05).

To clarify whether the exposure indeed does not lead to micronucleus induction even though mitotic disturbances had been reported under similar conditions, the micronucleus test and proliferation rate experiments were extended. In a separate set of experiments, cells were exposed for 24 h at a higher power intensity of 2 mW/cm^2^. Analysis was performed on a much higher number of cells, namely 30,000 for exposed and sham-exposed cells and 18,000 for controls. These exposures did not lead to increased micronucleus frequencies, whereas MMC treatment caused an increase in DNA damage in HaCaT (Fig. 5a) and HDF (Fig. 5b) cells. These experiments were also performed with A_L_ cells, because the mitotic disturbances had been investigated in these cells. Again, no increase in genomic damage in the form of micronucleus formation was observed as a consequence of the exposure (Fig. 5c).

**Figure 6:**
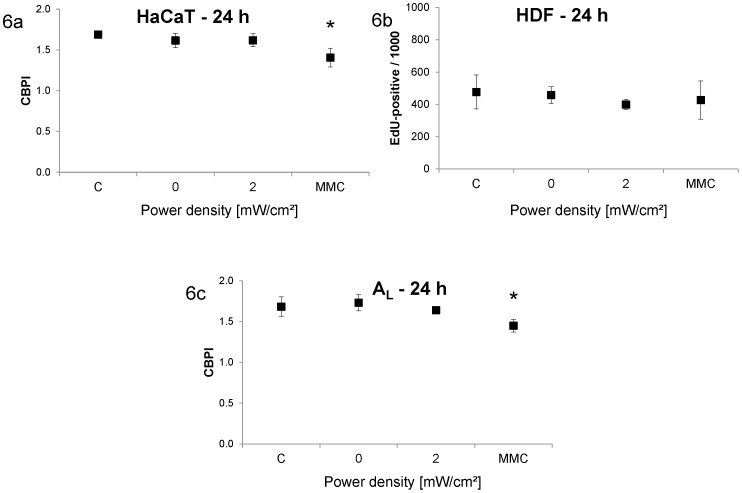
Proliferation rate after different exposure conditions. Columns represent means and error bars represent standard deviations of at least three independent experiments (at least 5×1,000 cells per replicate for exposed and sham-exposed samples; at least 3×1,000 cells per replicate for control samples). Results are shown as cytochalasin B proliferation index for HaCaT (6a) and A_L_ (6c) cells and as number of EdU-positive cells per 1,000 cells for HDF (6b) cells. MMC-treated HaCaT and A_L_ cells showed significantly lower proliferation indices compared to untreated cells (*, p<0.05).

Proliferation was also quantified for these experiments. No significant alterations, which could explain the lack of micronucleus induction, were detected (Fig. 6a–c).

## Discussion

Terahertz electromagnetic fields have not been investigated widely in terms of biological effects in the past, despite the increasing relevance due to new applications also involving human exposure to these electromagnetic fields. Only recently, some additional investigations on this topic have been published [15]. The majority of the studies published so far investigated effects at frequencies between 0.100 THz and 0.150 THz. This is not only due to the fact that sources and detectors are easier to handle at these frequencies, but also because future applications will most likely be using this frequency region. The currently employed types of body scanners, which are often associated with terahertz electromagnetic fields, are in fact using millimeter waves (0.03 to 0.10 THz) at the moment, but next generation scanners will likely work at higher frequencies including the terahertz range. Even though intensities are very low, these applications imply an exposure to a significant part of the general population, making it imperative to study putative biological effects.

One major contribution to this research field was the “THz Bridge” project [16], investigating mainly genotoxic effects in blood samples. Genotoxicity studies are of crucial importance because of the close link between genotoxic effects and carcinogenesis. In contrast to the THz Bridge studies, human skin cells were used as biological systems in the present study because terahertz electromagnetic fields cannot penetrate the human body deeply, making the skin the primary target organ of these fields. Human dermal fibroblasts (HDF cells) as primary cells and HaCaT cells, a keratinocyte cell line, were exposed with 0.106 THz with intensities below, at, and above the current safety limit of 1 mW/cm^2^.

The first test was the comet assay, which investigates DNA single and double strand breaks. For both cell lines no statistically significant induction of DNA migration after exposure to terahertz electromagnetic fields was observed in the comet assay compared to the respective sham controls. This finding is in line with other publications looking at DNA strand breaks in lymphocytes after exposure with 0.120 THz and 0.130 THz [21], [32].

The micronucleus frequency was also not affected by the terahertz exposure in both cell types. It was observed that the micronucleus frequencies differed clearly between the different cell types. This underlines the importance of investigating effects both on primary cells as well as on cell lines. These findings also confirm the results of other publications which found no increase in micronucleus formation caused by terahertz electromagnetic fields [18], [21], [32]. In contrast to this, aneuploidy, i. e. numerical chromosome aberrations not detectable as micronuclei, and mitotic disturbances were reported to be caused by terahertz electromagnetic fields at similar or lower power intensities [19], [20]. Both findings do not fit very well to the lack of micronucleus formation. As mitotic disturbances in particular are thought to develop to micronuclei, at least some of the disturbed mitoses would have been expected to form a micronucleus. Also, experimental exposure conditions like frequency and power intensity were similar for the finding of the mitotic disturbances in comparison to the present study. One hypothesis was that the electromagnetic field acts only on mitotic cells. Since for the analysis of mitotic disturbances cells are fixed directly after exposure, one can expect that all cells, which are analyzed, had been in mitosis during the exposure. With the standard micronucleus test protocol, cells are cultivated for a post-exposure incubation time with cytochalasin B to make micronucleus formation possible. This means that cells could have been in any cell cycle phase during exposure and only a small percentage of the analyzed cells had been in mitosis during exposure. To avoid this problem, cells were next exposed for 24 hours and fixed directly afterwards. Thus, all analyzed cells went through at least one complete cell cycle during exposure and hence must have been in mitosis. Cells were exposed with an intensity of 2 mW/cm^2^ and statistical power was raised by analyzing an increased number of cells, at least 10,000 for each repeat experiment for exposed and sham-exposed cells (yielding a total of at least 30,000 cells) and at least 6,000 for controls (yielding a total of at least 18,000 cells). Both for HaCaT and for HDF cells, no increase in micronucleus formation was observed. In a final step to adjust the protocol to the experimental conditions with which the mitotic disturbances were observed, the latter experiment was repeated with A_L_ cells, a human-hamster hybrid cell line, in which mitotic disturbances had been reported [20]. All other experimental parameters were kept constant. Again no change in micronucleus frequency was observed. This result confirms the first part of this study as well as other investigations [18], [21], [32], namely that terahertz electromagnetic fields do not cause direct DNA damage. It remains open whether the reported mitotic disturbances [20] or the aneuploidy induction [19] will be confirmed by independent investigations. In particular, the fate of the affected cells will have to be considered, since such rare events may be repaired by either correcting the problem before completion of mitosis or by eliminating the cell from the culture.

In conclusion, human skin cells were exposed to 0.106 THz electromagnetic fields and investigated for genotoxic effects. No induction of DNA strand breaks or chromosomal damage was observed. Very small alterations might not have been detectable because the cells showed considerable background level of DNA damage. Since mitotic disturbances had been reported to be caused by terahertz electromagnetic fields, the protocol for the micronucleus test was adapted. Again, no damage was observed. Contrary to the expected outcome, these mitotic disturbances do not seem to develop to manifest DNA damage.
